# ‘A certain magic’ – autistic adults’ experiences of interacting with other autistic people and its relation to Quality of Life: A systematic review and thematic meta-synthesis

**DOI:** 10.1177/13623613241255811

**Published:** 2024-06-03

**Authors:** Georgina Watts, Catherine Crompton, Catherine Grainger, Joseph Long, Monique Botha, Mark Somerville, Eilidh Cage

**Affiliations:** 1 University of Stirling, UK; 2 The University of Edinburgh, UK; 3 Scottish Autism, UK

**Keywords:** autism, autistic culture, double empathy, mental health, peer support, qualitative synthesis, Quality of Life, social communication, systematic review

## Abstract

There is an increasing focus on research exploring autistic communication and community. In this review, we systematically collate and analyse how autistic adults describe their experiences of other autistic people and the relationship this has with their Quality of Life (QoL). Fifty two qualitative papers were analysed using thematic meta-synthesis. Results found many experiences of other autistic people were positive and were associated with improved QoL across a range of domains. The heterogeneity of the autistic experiences described indicates a need for further research to explore which factors predict a positive impact on QoL. Analysis also highlighted a paucity of data on autistic people with co-occurring intellectual disability in relation to this phenomenon. Implications for research and practice are discussed.

**Lay abstract:**

Research has suggested that autistic people enjoy spending time with other autistic people and find them easier to talk to. We wanted to find out what autistic people say about spending time with other autistic people and whether this makes their life better. We found 52 papers which described this and reviewed what they found. We found that many autistic people had positive experiences of spending time with other autistic people and these experiences had positive impact on their lives in a range of different ways. The papers did not tell us whether this also happens for autistic people with a learning disability. More research is needed to find out more about why spending time with other autistic people helps some autistic people.

## Introduction

Autistic people often report negative social communication experiences which impact on areas of life, such as friendships (Billstedt et al., 2011; Seltzer et al., 2004), romantic relationships (Yew et al., 2021) and employment (Smith et al., 2014; Strickland et al., 2013). ‘Deficits’ in social communication remain a defining feature within autism diagnostic criteria (American Psychiatric Association, 2013). This deficit-based view of social communication has led to a vast research literature which sought to mitigate these perceived ‘deficits’ (Gernsbacher & Yergeau, 2019; Wattanawongwan et al., 2023). Interventions aimed to improve autistic social communication by teaching normative social skills, including making ‘appropriate’ eye contact during job interviews (Yoshikawa et al., 2023). Evidence for the efficacy of such approaches is limited (Bottema-Beutel et al., 2018; Morrison et al., 2020) and the underlying preconception of autistic social communication as defective non-autistic social communication has come under increased scrutiny in recent years (Davis & Crompton, 2021).

In contrast to deficit-based approaches, Milton’s Double Empathy Problem proposes social communication difficulties between autistic and non-autistic people are relational; autistic people struggle to understand non-autistic people, but non-autistic people also struggle to understand autistic people (Milton, 2012). Therefore, as communication breakdowns between autistic and non-autistic people are bidirectional, autistic people might communicate better with autistic people and non-autistic people better with non-autistic people.

A growing body of research aligns with this framework (Milton et al., 2022). Studies suggest autistic people experience better rapport (Crompton, Sharp, et al., 2020) and more effective communication (Crompton, Ropar, et al., 2020) when interacting with autistic people rather than non-autistic people. Autistic adolescents are more likely to initiate and reciprocate interactions with autistic than non-autistic peers (Chen et al., 2021).

**Figure 1.**
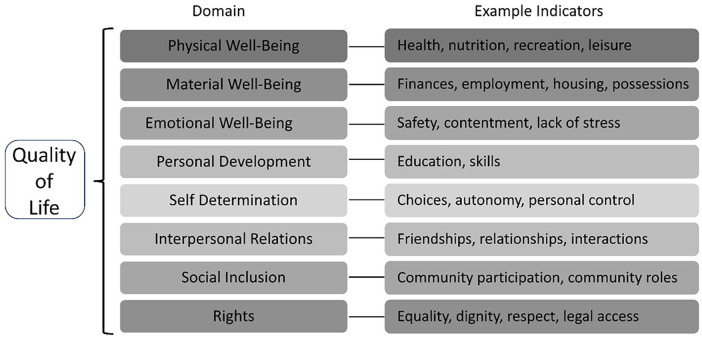
Quality of life conceptual model.

Differing understandings of autistic social communication influence how autistic social behaviours are interpreted within research and practice. Deficit approaches frame autistic differences as defective compared to non-autistic social communication styles, while a double-empathy interpretation focuses on the mismatch across neurotypes. Dominant deficit-based perspectives in research literature and public discourse contribute to stigmatisation of autistic people (Botha & Cage, 2022; Bottema-Beutel et al., 2021). While the role of stigma in well-being for marginalised people has long been recognised (Goffman, 1963), recent work has focused on the relationship between stigma and poorer Quality of Life (QoL, Holubova et al., 2016; Markowitz, 1998; Slater et al., 2015; Utsey et al., 2002). QoL is defined by the World Health Organization as ‘an individual’s perception of their position in life in the context of the culture and value systems in which they live and in relation to their goals, expectations, standards and concerns’ (The WHOQoL Group, 1998, p. 551). Measures of QoL have been used in intellectual disability research for over 50 years (Schalock, 2000). Schalock et al. (2008) divide QoL into eight domains, which we employ in assessing QoL outcomes in this review. Example indicators (see Figure 1) demonstrate how each domain is understood within Schalock’s QoL framework. These domains provide a holistic view of well-being compared to evaluating only some aspects of life such as social inclusion or mental health.

Autistic people typically have poorer QoL than non-autistic peers, and experience disadvantages across every QoL domain (Moss et al., 2017; Plimley, 2007). For example, within *Emotional Well-Being*, autistic people typically have poorer mental health than non-autistic peers (Lai et al., 2019), while barriers to education (Anderson et al., 2017) and employment (Ezerins et al., 2023) impact on *Material Well-Being*, *Personal Development* and *Self-Determination*. Historically, it was assumed these disadvantages (and thus impact on QoL) result from autistic deficits (Ezerins et al., 2023). This overlooks the possibility many disadvantages may be ascribed to societal factors like stigma (Grinker, 2020; Turnock et al., 2022), which create barriers to better QoL.

Studies indicate community contact may benefit QoL for autistic people. Contact with other autistic people provides access to narratives challenging deficit-based perceptions of autistic personhood facilitating positive autistic social identities (Kapp et al., 2013). Increased identification with a positive autistic social identity has been associated with better mental health and may mediate the impact of stigma on well-being (R. Cooper et al., 2021).

Furthermore, successful communication experiences (Crompton, Ropar, et al., 2020) and rapport (Rifai et al., 2022) between autistic people may have a positive impact on QoL (Black et al., 2022). Distinctly autistic social communication styles may facilitate communication and friendships between autistic people (Heasman & Gillespie, 2019), benefitting QoL domains including *Social Inclusion* and *Interpersonal Relations*.

Awareness of autistic communities and culture is increasing (Kapp, 2020). Outside of the literature, autistic people often describe contact with other autistics as positive, suggesting that friendships with other autistic people are less socially demanding and allow people to be their authentic selves (Forbes, 2021; Quincy, 2019). While research into this phenomenon is limited, initial studies suggest contact with other autistics may benefit mental health and social interaction (Botha et al., 2022; Crompton, Hallett, et al., 2020). Both autistic-focused literature and research on other minority groups suggest value in exploring the role of community contact for autistic QoL. The purpose of this review is to systematically collate and analyse literature to answer the question: how do autistic adults describe spending time with other autistic people and how does this relate to Quality of Life?

## Methodology

This review was registered on PROSPERO (registration number CRD42023393210) and followed Preferred Reporting Items for Systematic Reviews and Meta-Analysis (PRISMA) guidelines (Page et al., 2021).

## Search strategy

As the research question sought rich descriptive data about autistic experiences only qualitative research papers were included in this review. Authors, with support from University librarians, developed a search strategy to identify papers reporting data on autistic adult contact with other autistic people and how this related to QoL. In order to avoid making predictions about where the phenomenon might be expected to occur, we sought descriptions of autistic adults’ experiences of other autistic people that took place in any context with any other autistic person, including with autistic children.

We searched Cochrane and PROSPERO for similar reviews but found none. Key papers were used to test search strings and inform search strategy revisions. Search strings included terms relating to autistic people; contact with other autistic people; and QoL (see Appendix A). We conducted electronic searches on Scopus, PsycINFO, PsycArticles and Web of Science, with additional searches on Proquest and Web of Science to identify relevant grey literature such as unpublished theses and conference proceedings.

## Review criteria

Studies meeting the following criteria were included:

(a) Reports data on autistic adults (aged 18 or over) with or without any other co-occurring condition(s);
(b) Includes data on autistic adults’ experiences of any other autistic people (including autistic children) in any setting;
(c) Includes data on how autistic people’s experiences of other autistic people relates to their Quality of Life;
(d) Reports qualitative data;
(e) In English.

Papers published in a peer reviewed journal were included. In addition, grey literature including unpublished theses and conference proceedings were included.

## Study selection

**Figure 2.**
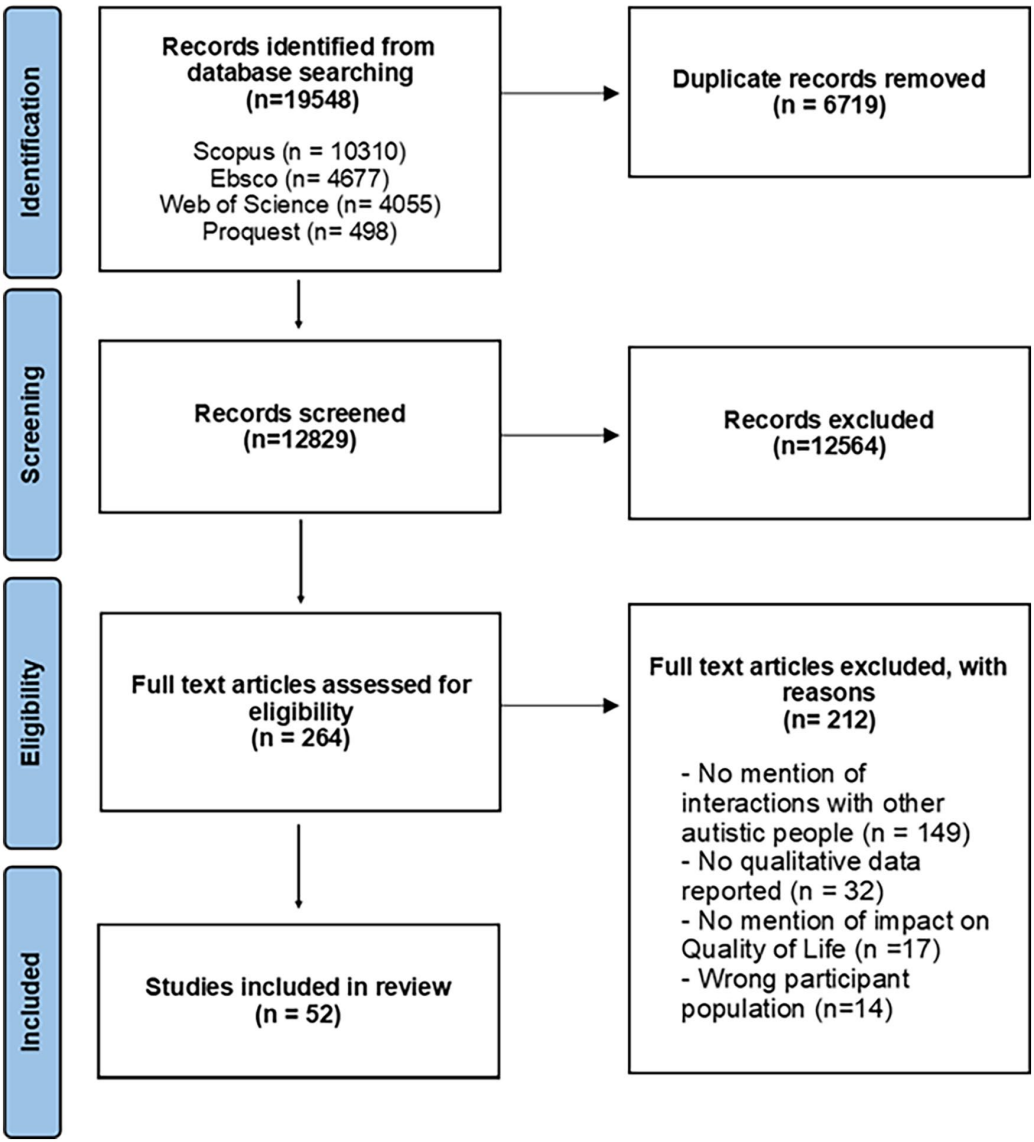
PRISMA flow diagram.

Database searches conducted on 29 August 2022 resulted in 19,548 identified records (see Figure 2) which were uploaded to Covidence systematic review software. 6719 duplicates were removed and titles and abstracts of the remaining 12,829 records were screened by a reviewer (GW). A second reviewer (MS) independently screened 10% (*n* = 1283) of titles and abstracts, resulting in 1266 agreements (98.67%, κ = 0.63). Disagreements were resolved through discussion between two reviewers (GW and MS).

A reviewer (GW) screened the full text of the remaining 272 records. A second reviewer (MS) independently screened the full text of 10% (*n* = 27) of the remaining records, 5 conflicts between reviewers resulted in lower agreement than expected (81.48%). Discussion between GW and MS identified discrepancies in the application of inclusion criteria. Conflicts were resolved through discussion. MS screened a further 13 records which resulted in an improved agreement (92.50%, *k* = 0.81). Unresolved conflicts were discussed with other research team members (EC/CG/JL/MB) until concordance was achieved. Fifty-two records were eligible for inclusion.

## Data extraction

One reviewer (GW) conducted data extraction for all eligible studies in Covidence (Covidence Systematic Review Software, n.d.). A second reviewer (MS) extracted data from 12% of studies (*n* = 6). Although inter-rater reliability was not calculated, agreement between reviewers was very high (estimated 85%) and disagreements were resolved through discussion between reviewers.

## Quality assessment

Study quality was analysed using Critical Appraisal Skills Programme Qualitative Studies Checklist (CASP, UK, n.d.), which is designed to assess methodological strengths and limitations (Hannes & Macaitis, 2012). This was applied by one reviewer (GW) with 12% (*n* = 6) analysed by a second reviewer (MS). In accordance with Cochrane Handbook guidance (Higgins et al., 2019), the CASP was not scored, but guided evaluation of limitations on study rigour and implications for the review (Noyes et al., 2019).

## Data synthesis

Data were analysed using qualitative meta-synthesis, which allows qualitative studies to be interpreted into abstract analytical themes which go beyond original study findings (Erwin et al., 2011). Data from ‘results’ and ‘discussion’ sections of included papers, including both participant quotes and author interpretations, were entered verbatim into NVivo, before the three stages of synthesis were applied (Thomas & Harden, 2008). First, data were analysed inductively using line-by-line coding to create new codes, with a second reviewer (CG) coding 10% of papers (*n* = 5) resulting in high agreement (85%) and conflicts resolved through discussion. Second, codes were grouped into descriptive themes through identification of similarities and differences. Third, descriptive themes were used to inform interpretative analytical themes.

## Community involvement

The first (GW), fifth (MB) and sixth (MS) authors are autistic researchers. There was no other autistic community involvement in this review.

## Author positionality

The first (GW), fifth (MB) and sixth (MS) authors are self-advocates involved with the autistic community. The remaining authors (EC, CC, CG and JL) are non-autistic autism researchers. All authors view autistic people from a neurodiversity-affirming standpoint aligned with the social model of disability. Autistic and non-autistic authors worked together to reflect on preconceptions about autistic people which might influence this work.

## Results

### Participant characteristics

The 52 studies included in this review involved 2872 participants. Demographic information is reported in Table 1.

**Table 1.** Participant demographics.

Demographic	Number of studies^1^ in which demographic were reported	Categories	Value
Neurotype	48 (*n* = 2872)	Autistic^2^	*n* = 2615
		Non-Autistic	*n* = 257
Age (years)	43 (*n* = 2752)	Range	12-78
	31 (*n* = 2495)	Mean	33.31
Gender^3^	46 (*n* = 2817)	Male	*n* = 924
		Female	*n* = 1699
		Non-Binary	*n* = 78
		Transgender	*n* = 42
		Other gender identities^4^	*n* = 63
		Prefer not to say	*n* = 11
Diagnostic Status	45 (*n* = 2830)^5^	All participants clinically diagnosed	*n* = 1875
		Mixture of clinically diagnosed or self-identified	*n* = 955
Ethnicity	26 (*n* = 1121)	Caucasian	*n* = 860
		Mixed race	*n* = 66
		Black	*n* = 33
		Asian	*n* = 31
		Hispanic	*n* = 26
		Other	*n* = 26
		Prefer not to say	*n* = 4

1 Four papers (Large & Serrano, 2018; Mantzalas et al., 2022; Milton & Sims, 2016; Nagib & Wilton, 2021) analysed secondary data and were not able to report demographic data, therefore the maximum number of papers possible here is 48.

2 Including self-identified autistic participants.

3 Gender refers to gender identity. Where some papers reported both gender assigned at birth and gender identity only gender identity is included here.

4 ‘Other gender identities’ includes participants who described their gender identity as gender fluid, gender queer, two-spirit, agender, and no gender. Where some papers grouped several genders together (e.g. ‘gender fluid, gender queer or nonbinary’) these were also included within the Other gender identities category.

5 Some studies described participants as autistic but did not report their diagnostic status.

Most studies reported age and gender data (Table 1), though fewer reported characteristics like sexuality, education and co-occurring conditions. Eighteen studies (34.62%) included some participants who did not meet inclusion criteria for this review, e.g. autistic children and young people, or non-autistic neurodivergent people. Where possible (i.e. papers which included participants’ age or diagnostic status alongside quotes) we extracted only data relating to autistic adult participants. Given the paucity of research on this topic, for papers that did not clarify whether specific quotes were given by autistic adult participants or another (non-autistic or child) participant (*n* = 6), all quotes were included. This was done to ensure that all autistic voices could be heard as part of this research.

### Study characteristics

Studies were published between 2012 and 2022, and represent research from the UK (23), USA (13), Australia (6), Canada (5), Belgium (3), Israel (1) and Sweden (1).

Seven studies (13.46%) explicitly aimed to explore interactions between autistic people, while the remaining 45 (86.54%) focused on a diverse range of subject areas such as the experiences of autistic parents, Covid-19, employment and victimisation.

Only 13 (25%) studies reported making adaptations to increase accessibility of participation, and only six (12%) included participants who had a co-occurring intellectual disability, were non-speaking or had high support needs. Within these, only seven participants with an intellectual disability are reported (from two studies) while a further 10 participants are described as non-speaking and/or having high support needs within the remaining studies. Therefore, across all studies, the total number of participants who either had an intellectual disability, were non-speaking or had high support needs (*n* = 17) represents 0.006% of all participants represented (*n* = 2872).

### Quality appraisal

The CASP was used to evaluate study quality, though scores were not calculated and did not determine whether studies were included in the review (Higgins et al., 2019). Of particular note was question 6, which asks whether the relationship between researcher and participants has been adequately considered. Only 21 papers (40.38%), reported information meeting criteria for this question such as reporting on positionality or community involvement.

### Thematic meta-synthesis

**Figure 3.**
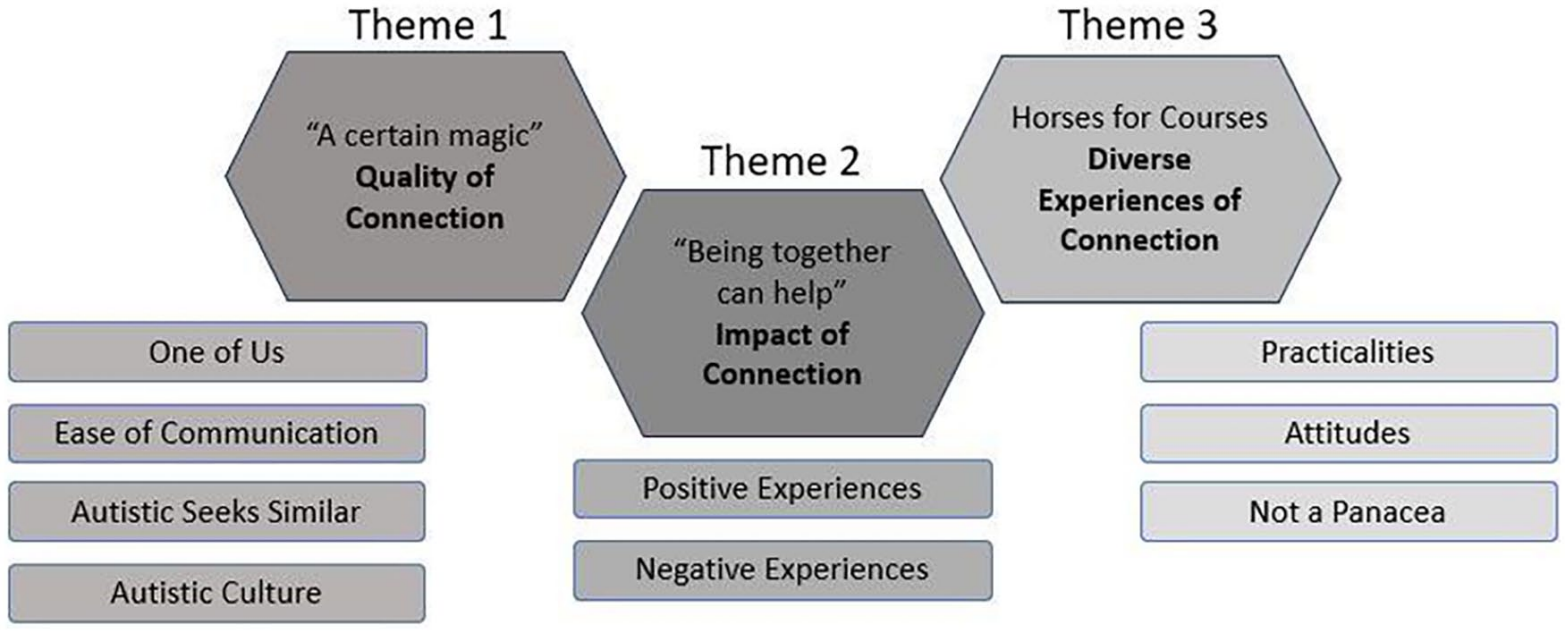
Overview of themes and subthemes.

Overall, three superordinate analytic themes (with corresponding subthemes) were generated: Theme 1: ‘A certain magic’ – Quality of Connection; Theme 2: ‘Being together can help’ – Impact of Connection; Theme 3: Horses for Courses – Diverse Experiences of Connection, each with several subthemes (Figure 3). These are described below, with associated QoL domains in italics.

### Theme 1: ‘a certain magic’ – quality of connection

The magic referred to in this theme title reflects the idea something special occurs within autistic-autistic interactions; ‘when two autistic people are together in the same room or even linked by written communication, a certain magic is created, like, electricity in the air’ (Schneid & Raz, 2020, p. 5). This is relevant to *Social Inclusion* and *Interpersonal Relations* QoL domains – different aspects of this phenomenon are captured by four subthemes.

### Subtheme 1: ‘*one of us*’

Being ‘One of us’ reflects a sense of shared identity and belonging within a group expressed across multiple papers. Autistic people recognised themselves in others and shared experiential knowledge, meaning they felt understood and accepted. Schneid and Raz (2020) described how all of their autistic participants ‘said that autists would understand them better’ (p. 5).

Mutual understanding between autistic people created a sense of closeness. Many papers echoed experiences described by Botha and Frost (2020) as ‘The space among autistic people was presented as safe, validating and supportive’ (p. 8). Acceptance and shared understanding with other autistic people were associated with an increased sense of belonging:

As lovely as all my neurotypical friends are, I feel I belong there [with autistic people], and I am like everybody else. I have never had that before . . . I feel like I understand people and they understand me. (Crompton, Ropar, et al., 2020, p. 1444)

This sense of *Social Inclusion* contrasted with previous experiences of interactions with non-autistic people, with many describing joy on discovering “wow, there are others like me!” (Mattys et al., 2018, p. 328) and a sense of relief: “Finally I’m not alone” (Tan, 2018, p. 166).

### Subtheme 2: ‘*ease of communication*’

Many participants said *Interpersonal Relations* with other autistic people were easier than with non-autistic people. ‘Ease of communication’ was frequently described as speaking the same language: ‘With autistic people, who speak my language . . . it goes fantastically well most of the time’ (Livingston et al., 2019, p. 771). Participants believed ‘communication styles were similar between autistic people, and this made interactions more comfortable that it was easier to follow conversations and understand what people mean’ (Crompton, Hallett, et al., 2020, p. 1443), which meant ‘interactions with autistic people were less effortful and tiring’ (Cummins et al., 2020, p. 684).

Participants emphasised the facilitating role of acceptance: ‘Being in these comfortable and accepting environments ‘allows [them] to relax [and] be able to communicate effectively and honestly’’ (Howard & Sedgewick, 2021, p. 2272).

### Subtheme 3: ‘*autistic seeks similar*’

Consistently across studies, many participants sought out opportunities to interact with other autistic people. Schneid and Raz (2020) described how ‘Respondents emphasized that they wanted friends who are like them’ (p. 6) while Pearson et al. (2022) stated ‘Autistic only spaces are desperately needed more than anything else right now’ (p. 8).

The desire for contact with other autistics was often framed in terms of shared identities:

I was excited when I first got my diagnosis. I felt I just want to meet other people like me. It’s like I’ve been an animal with spots. All my life I’ve been around other animals without spots and I feel I can start meeting other people like me with spots, you know? (Tan, 2018, p. 166)

Some also described a desire to spend time with autistic people who also shared other intersectional identities. Leedham et al. (2020) described how some autistic women ‘found comfort in identifying with others within the female autism community’ (p. 143) and papers which focused on other autistic populations (such as LGBTQIA+) described a similar desire to be with those who shared their marginalised identities (Strang et al., 2021). Some participants wished they had experienced *Social Inclusion* with other autistic people at a younger age:

all of the adults reported that social participation with those who understand them and who are similar to them, including people with ASD, as significantly impacting their QOL. Many of the adults reported that they would have benefitted from this type of social participation as a child and adolescent. (Pfeiffer et al., 2016, p. 7)

### Subtheme 4: ‘*autistic culture*’

Autistic ways of being together feature distinct communication styles and social conventions which may be described collectively as Autistic Culture. Experiences of being in autistic cultural spaces give rise to the ‘magic’ described in *Interpersonal Relations* between autistic people and was often distinguished from relationships with non-autistic people: ‘There is absolutely nothing like meeting another autistic person and relating to them in the unique way that we do. You can’t have that with anyone else, autistic people just understand, no explanation necessary’ (Oredipe et al., 2023, p. 7). Some even described being able to sense other autistic people: ‘When I meet my people, I know, because I feel it’. (Milton & Sims, 2016, p. 531)

Shared experiences and improved communication created opportunities to increase *Social Inclusion*: ‘One participant summed up time with autistic friends as “No masking. All fun!” Others echoed this sentiment, saying “thank god for finding my tribe” because other autistic people did not judge them for autistic behaviours’ (Howard & Sedgewick, 2021, p. 2272).

Some papers explicitly described distinctively autistic cultural conventions: ‘Some participants felt that they had created ‘[their] own societal rules within our home’’ (Howard & Sedgewick, 2021, p. 2272). Others alluded to differences between autistic and non-autistic social conventions describing unconventional actions which might not reflect neuro-normative expectations but nevertheless work for them:

I’ve made some friends there [at Autscape] . . . we rarely see each other outside of scheduled meetings . . . sometimes we will talk over the internet . . . it’s probably not quite how other people would see a friendship but I’d get very overwhelmed if they wanted to talk all the time. (Botha et al., 2022, p. 6)

### Theme 2: ‘*being together can help*’ – impact of connection

People on the spectrum struggle in different ways and overcome things in different ways, but *being together can help* [emphasis added] . . . I spoke to people on the spectrum and I have given them advice and they have given me advice (Crompton, Hallett, Axbey, et al., 2022, p. 9)

Theme 2 documents outcomes of contact with other autistic people across many QoL domains. The title quote (‘Being together can help’), reflects the ‘Positive Experiences’ described in many papers, yet the ‘Negative Experiences’ subtheme demonstrates positive impacts are not universal.

### Subtheme 1: ‘positive experiences’

Many papers suggested ‘being together can help’ *Emotional Well-Being*. Participants attributed better mental health to having opportunities to learn from others. This included opportunities to ‘share helpful strategies’ (Crompton, Hallett, Axbey, et al., 2022, p. 9), and emotional support gained from talking to peers with similar experiences; ‘It’s not so embarrassing if it is a shared experience, if I say or do the wrong thing’ (Siew et al., 2017, p. 10).

Some participants described generally positive impacts on *Emotional Well-Being* such as improved self-esteem, while others gave more emphatic descriptions contrasting benefits of connection with extreme distress experienced prior to finding autistic communities:

Codey reported feeling suicidal prior to finding ASD-positive communities and four other participants expressed having ideated or attempted suicide prior to receiving an ASD diagnosis. After acquiring an ASD diagnosis, participants used the label to locate autistic communities, which expanded their social networks and contributed to a more positive concept of self. (Tan, 2018, p. 166)

Being with other autistic people provided opportunities to increase self-knowledge and *Personal Development*: ‘Part of social connectedness was giving and receiving advice, allowing the opportunity to learn about oneself’ (Botha et al., 2022, p. 6). This identity development contrasted with self-stigma, as described by Seers and Hogg (2022): ‘This self-blame was released once a diagnosis was obtained and participants connected with other autistic women, thereby taking an active role in their identity development’. (p. 6). A more positive autistic social identity was associated with being with other autistic people who can ‘challenge the normative, deficit understanding of autism’ (Seers and Hogg, 2022, p. 13). Seeing autistic role models flourish helped people feel more optimistic about their future: ‘I appreciated being in [dis]course with people on the spectrum who have been married and people who have children. I had a lot of hopelessness previously because I didn’t know if these things were possible for me’. (Rothman et al., 2022, p. 10).

Beyond impact on *Emotional Well-Being*, papers also described positive impacts across all QoL domains in a variety of ways. Some studies created peer groups to facilitate specific outcomes, e.g. the training and employment peer support group which ‘enabled adults with HFASD to feel more confident to enter the workplace’ (Southby & Robinson, 2018, p. 514) which is relevant to *Material Well-Being* and *Personal Development*. In other instances, the context of interactions naturally facilitated certain outcomes such as the political connectedness described by Botha et al. (2022) which ‘allowed people a chance to challenge stigmatising representations and narratives’ (p. 10) and suggests a positive impact on *Self-Determination* and *Rights*.

Some participants described general improvements such as learning to cope better in daily life which has bearing on all aspects of QoL. Others described specific outcomes, relevant to a range of QoL domains, including feeling able to start dating again (Rothman et al., 2022, *Interpersonal Relations*); feeling able to disclose as autistic to friends (Goddard & Cook, 2022, *Rights*, *Social Inclusion* and *Interpersonal Relations*); feeling empowered to apply for and achieve employment (Wallace, 2016, *Material Well-Being*, *Personal Development*); potential for peer support to impact on educational outcomes (Crompton, Hallett, Axbey, et al., 2022, *Personal Development, Self-Determination*); increased likelihood and motivation to join other clubs (Wallace, 2016, *Social Inclusion*, *Interpersonal Relations*); and a participant who ‘suggested that attending an autism social group could potentially reduce his need to visit the GP’ (in the UK a GP is a doctor who provides general medical treatment, Courts, 2022, p. 208, *Physical Well-Being*).

### Subtheme 2: ‘negative experiences’

Some contact with autistic people had a negative impact. First, some participants experienced conflicting sensory needs with other autistic people, especially where their ‘own autistic needs clashed with their child’s needs’. For others, conflicting needs related to social communication styles, finding it difficult ‘When one person talks too much’ (Gillespie-Lynch et al., 2017, p. 10).

Second, providing peer support took its toll: ‘If you are being the person that is taking an emotional burden from somebody then you need somewhere to put it as well’. (Crompton, Hallett, McAuliffe, et al., 2022, p. 9). Within family contexts, improved understanding of their children due to their shared neurotype created pressure where parents ‘had to explain or ‘translate’ (Alice) difficulties to others, or ‘mediate’ (Alice) conflicts’ (Dugdale et al., 2021, p. 1977). Some autistic parents described an extremely negative impact of parenting autistic children: ‘The extent of these feelings is depicted in their choice of words: “Like Beirut,” “a war zone,” “a hostage” and “trapped”’. (Marriott et al., 2022, p. 3187). However, this article also comments that the literature suggests such experiences are common across all parents of autistic children, not only autistic parents.

Finally, some participants described the negative impact of vicarious struggle. Parents were described as ‘deeply affected by having to bear witness to their child having similarly difficult childhood experiences to their own’ (Marriott et al., 2022, p. 3187). Contrasting with hopefulness created by seeing others’ success, seeing others’ distress increased concerns for the future, and reminded participants of their own difficulties. Henley (2021) ‘found the group autism sessions really harrowing. I was one of eight people who attended, all men. I found some of the stories the others told heart breaking to listen to’. (p. 259).

### Theme 3: horses for courses – diverse experiences of connection

The idiom ‘Horses for Courses’ means different things suit different people and is used here to reflect the diversity of autistic experiences. Theme 3 describes different aspects of this diversity and considers the factors which influence connection. These factors are not described in relation to specific QoL domains but rather highlight the wide range of variables impacting the accessibility of QoL outcomes described in the preceding themes.

### Subtheme 1 – ‘practicalities’

Several factors influenced accessibility of contact with other autistic people, including physical, social and communicative accessibility and compatibility of individual needs with available contact.

Sensory accessibility, as well as mobility, was a key component of accessibility; some people avoided meetings in noisy venues. Some saw online interactions as more accessible as they could control the sensory environment. Location was an important barrier for some people, for example, ‘not being able to drive to unfamiliar places’ (Marriott et al., 2022, p. 3188).

Social accessibility determined whether people were able to engage with others. Crompton, Hallett, Axbey, et al. (2022) reported peer support group size preferences varied and familiarity with others in the group influenced these preferences. One participant described frustration that local opportunities to meet other autistic people were socially inaccessible: ‘It is like going to a pub to meet a load of people you don’t know, is like my worst nightmare. So I have never done it’. (Crompton, Hallett, McAuliffe, et al., 2022, p. 9).

Lack of diversity created barriers to community. Autistic people who have other marginalised identities often desire connection with autistics who share these identities. One paper described how ‘there are other things that non-white autistic people are party to that white autistic people wont understand’ (Crompton, Hallett, McAuliffe, et al., 2022, p. 8), while another said ‘almost all the regular attendees were male and engaged in similar interests, and this was off-putting to a female participant who stopped going as a result’. (Goddard & Cook, 2022, p. 2708).

Online contact improved accessibility as it allowed communication ‘without the pressures associated with face-to-face communication’ (Trembath et al., 2012, p. 221), and ‘time to order my thoughts and edit what I am saying’ (Seers & Hogg, 2022, p. 13).

A lack of in-person groups was the biggest barrier: ‘I felt alone because I didn’t know any other autistic people and there weren’t any support groups at my community college’. (Oredipe et al., 2023, p. 8). Some papers reported groups being ‘discontinued due to a lack of funding, space, or someone to coordinate’. (Crompton, Hallett, McAuliffe, et al., 2022, p. 9) and concern about short-term availability of groups:

Once we had completed the eight 2 h sessions, we were informed that was the end of this process and that there was no further support locally for us. I started to feel very concerned about the ongoing plight of those adults with autism, who it now appeared were right to think there was nothing in terms of ongoing support available to them. (Henley, 2021, p. 259)

### Subtheme 2 – ‘attitudes’

Participants expressed wide-ranging attitudes towards contact with other autistic people. While most described positive attitudes some had neutral or negative attitudes. Papers attributed discrepancies between these attitudes to two inter-related factors: knowledge and stigma.

For some, a lack of insight into the potential for positive experiences created a barrier to connecting with others, they ‘didn’t really see the point in trying to talk to other [autistic] people’ (Botha et al., 2022, p. 9). Others had stereotyped preconceptions around autistic sociality e.g. ‘it’s a social group and I thought that’s a bit weird because you know after reading up everything . . . social group for ASD that seems a bit odd but . . . that was a thing’ (Courts, 2022, p. 158).

Several studies described negative attitudes based on internalised stigma; people sought to distance themselves from a stigmatised identity. This resulted in downwards social comparisons with other autistic people, such as the participant in Botha et al. (2022) who said:

I’m extremely intelligent and joined MENSA but I’m not like other autistic people [. . .] Also there is a worry that what if they are not high functioning people and I couldn’t relate to that so. I mean, the other people with autism, smell is a big problem because a lot of them aren’t very hygienic . . . I couldn’t stand to be near them at all (p. 8)

Several studies suggested this internalised stigma may be mediated by increased connection with autistic community. One participant described the impact of learning from other autistic people: ‘It seemed like this whole huge collection of different things that were fucked about me’ . . . ‘And all of a sudden, I recognised that there were other people who had the same constellation of differences . . .’ ‘Now, I’m a normal autistic person, not an abnormal neurotypical’. (Tan, 2018, p. 164)

### Subtheme 3 – ‘not a panacea’

Several papers warn contact with other autistic people should not be viewed as a panacea for three reasons.

First, the heterogeneity of autistic experiences of other autistic people means no single approach to facilitating contact will be appropriate or beneficial for everyone. Mattys et al. (2018) summarise this variation of experience as being ‘either a big success (“finally I found others who think like me”) or a complete failure (“they have nothing in common with me and annoy me”)’ (p. 328). Thus, positives or negative impacts are not universal.

Second, the quality and impact of connection is not necessarily specific to the neurotype of interaction partners. Several papers described positive experiences with both other autistic people and supportive non-autistic people. Thus, some suggest positive outcomes may be contingent on quality of interaction rather than neurotype:

The opportunities created by AU magazine itself, Autscape and other such autistic-led spaces are obviously beneficial to many, yet so are relationships that accept and celebrate one’s way of being in the world, wherever such relationships are fostered and nurtured. (Milton & Sims, 2016, p. 534)

Third, peer support should not be the only option available to autistic people, who may also require mental health care from specialist providers. There are significant barriers to for autistic people accessing mental healthcare (Adams & Young, 2021; Coleman-Fountain et al., 2020): while peer support may play an important role in mental health and well-being, it will not meet everyone’s needs: ‘Peer support frameworks should not be used in place of specialist support’ (Crompton, Hallett, McAuliffe, et al., 2022, p. 9). Relatedly, some papers clarify that benefits of autistic-autistic contact should not be suggestive of segregation from society:

It’s very important to have autistic space for people . . . sometimes people fear this is a form of self silo-ing or segregation and I’m not trying to say we don’t need to survive in the non-autistic world too . . . but it’s such a lifeline for many of us. (Crompton, Hallett, et al., 2020, p. 1445)

## Discussion

This systematic review examines experiences of autistic adults’ contact with other autistic people and the relationship between this and QoL. Our results align with both informal reports from the autistic community and recent research grounded in neurodiversity-affirming approaches which challenge deficit-based approaches seen elsewhere in the literature. This pattern of alignment (and contrast) with the wider research is repeated across our results and has particular relevance for the QoL domains of *Interpersonal Relations; Social Inclusion*; and *Emotional Well-Being*.

As described elsewhere, *Interpersonal Relations* are contingent on the quality of interactions with others–communication between autistic people is often more successful and less stressful than communication between autistic and non-autistic people (Crompton, Ropar, et al., 2020; Heasman & Gillespie, 2019; Rifai et al., 2022). This challenges the common presumption that quality of autistic *Interpersonal Relations* is contingent on normative social communication skills, suggesting autistic social communication styles should be understood as different rather than defective.

Our results suggest that shared experiences and identities cultivated a sense of community and belonging between autistic people. This contrasted with prior experiences where misattunement and miscommunication contributed to loneliness. Other studies suggest contact with other autistic people may reduce loneliness (Elmose, 2020; Grace et al., 2022). Similarly, Botha and Frost (2020) suggest community connectedness may buffer the impact of Minority Stress on *Emotional Well-Being*. This aligns with findings from other disabled populations showing within group contact increases *Social Inclusion*, mitigating some impact of stigma (Daley et al., 2018; Salmon, 2013). This review showed a similar relationship where contact with other autistic people was described as counteracting the impact of stigma (and self-stigma) and having a positive impact on mental health.

We found autistic people learned about being autistic from other autistic people, helping them craft a more positive autistic social identity. Acceptance and validation from other autistic people facilitated a more positive self-image contrasting with stigmatising perspectives. Interactions between identity, stigma and *Emotional Well-Being* are also seen in research which suggests positive autistic social identity influences autistic mental health (K. Cooper et al., 2017). The evidence from this review and resonance with other research suggests contact with other autistic people may be key to increasing *Social Inclusion*.

The review also indicated some potential benefits to QoL domains not previously considered in relation to this phenomenon. These novel findings are of particular interest as they suggest that, for at least some autistic people, the benefits of contact with other autistic people may have a significant impact on aspects of QoL beyond those previously documented. Studies described participants feeling more able to seek and retain employment as a result of growing confidence. Such employment outcomes are relevant to the QoL domains of *Material Well-Being*, *Self-Determination* and *Personal Development* and resonate with research which suggests employment as a predictor of better QoL outcomes for autistic people (Mason et al., 2018; Walsh et al., 2014). Where better communication facilitated knowledge exchange, autistic people were empowered to self-advocate. The development of self-advocacy skills seen in the reviewed papers has implications for autistic people’s *Rights* which subsequently impacts on all aspects of QoL. Other research has also described self-advocacy as a key component of Self-Determination for autistic people and drawn similar conclusions about the impact of this on broader QoL outcomes (Kim, 2019). The present review’s findings also suggested that learning from each other meant that some participants were more able to access a range of facilities such as leisure, education and healthcare facilities (*Physical Well-Being; Self-Determination; Personal Development, Rights*). Again, the relationship between access to such facilities and QoL outcomes, as also seen in Schalock’s QoL indicators (Schalock et al., 2008, Figure 1) is born out in the research on predictors of autistic QoL (Chiang & Wineman, 2014; Mason et al., 2018). Therefore, the diversity of unanticipated benefits to QoL which are attributed to contact between autistic people by the papers in the present review, alongside the implications of these findings for practices which seek to improve autistic QoL, highlights the need for more research into this phenomenon.

### Limitations

While the reviewed studies include a range of research topics, they do not represent the entire autistic population. The lack of representation of autistic people with intellectual disability means we cannot extrapolate meaningful conclusions about this population. Similarly, the sparsity of other demographic data reported by studies limits the applicability of these findings to other intersectional autistic populations. For example, the low number of studies reporting ethnicity and high proportion of Caucasian participants in these studies may reflect broader racial disparity within autism research (Jones & Mandell, 2020).

In addition, the limited number of studies reporting whether researchers critically examined their own role and potential bias when conducting the study suggests a lack of awareness of the need for reflexivity and may indicate an increased risk of researcher bias.

### Implications

These results add to demands for research to reconsider deficit-based approaches to understanding autistic people. Implications of adopting less stigmatising perspectives within research are far reaching for both research and practice (Bottema-Beutel et al., 2021). It is vital for future research to consider the gaps in research knowledge identified here, primarily, the lack of research relating to autistic people who have an intellectual disability, are non-speaking or are described as having high support needs. The literature on social support in other marginalised populations (e.g. sexual minorities, Frost et al., 2016) suggests that the positive effect of intra-community connectedness is not a phenomenon that is unique to autistic people, further research is needed to evaluate to what extent research on other populations may map onto autistic experiences.

While more research is needed to explore which factors determine QoL outcomes, the clear desire of many autistic people to spend time with others, alongside the benefits described in these papers, evidences the need for more opportunities for contact between autistic people. In addition to demands for services to provide ongoing, accessible peer support opportunities, other approaches may also be useful such as signposting to autistic-led organisations and social media. The warning that informal peer support should not be seen as a substitute for other kinds of more formalised support is also seen in Cage et al. (2024) who found that while autistic people wanted peer connections, they also wanted access to high quality information. Therefore, it is important to ensure service providers and policy makers understand that providing opportunities for contact between autistic people is not a panacea.

## Conclusion

This is the first systematic review investigating the experience of autistic contact with other autistic people and the implications of this contact for QoL. The notable similarity of experiences across many different life contexts suggest these experiences can be significant and transformative for many autistic people. While autistic-autistic contact should not be seen as a panacea, these findings suggest contact between autistic people has the potential to improve QoL.

## Supplemental Material

sj-docx-1-aut-10.1177_13623613241255811 – Supplemental material for ‘A certain magic’ – autistic adults’ experiences of interacting with other autistic people and its relation to Quality of Life: A systematic review and thematic meta-synthesis Supplemental material, sj-docx-1-aut-10.1177_13623613241255811 for ‘A certain magic’ – autistic adults’ experiences of interacting with other autistic people and its relation to Quality of Life: A systematic review and thematic meta-synthesis by Georgina Watts, Catherine Crompton, Catherine Grainger, Joseph Long, Monique Botha, Mark Somerville and Eilidh Cage in Autism
