# Supplementary material for: ‘A certain magic’ – autistic adults’ experiences of interacting with other autistic people and its relation to Quality of Life: A systematic review and thematic meta-synthesis
Source: Autism. 2024 Jun 3;29(9):2239–53. doi: 10.1177/13623613241255811 (PMC12332227; doi:10.1177/13623613241255811)
Supplement: sj-docx-1-aut-10.1177_13623613241255811 – Supplemental material for ‘A certain magic’ – autistic adults’ experiences of interacting with other autistic people and its relation to Quality of Life: A systematic review and thematic meta-synthesis [file sj-docx-1-aut-10.1177_13623613241255811.docx]

Appendix A

Search string:

(autis* OR asperger* OR "ASD" OR "ASC") AND ("autistic community" OR peer OR "autistic pairs" OR "autistic-autistic" OR contact OR “autistic to autistic” OR “other autistic” OR connect* OR belonging OR coproduction OR "co-production" OR dyad OR participatory OR friend* OR relationship OR rapport OR community OR relation* OR "interact*") AND (wellbeing OR "well-being" OR "quality of life" OR "mental health" OR “life satisfaction” OR depress* OR anxi* OR wellness OR "self-esteem" OR happy OR "joy*" OR “thriv*”)

Database search strategy:

| **Database** | **Subsection of database** | **Grey Literature Search** | **Filters** | **Number of results** |
| --- | --- | --- | --- | --- |
| SCOPUS | (1788 to present) | No | Title-abs-key | 10318 |
| EBSCOhost | PsycInfo (1806 to present) | No | Title-abs-subject terms | 4677 |
|  | PsycArticles (1894 to present) |  |  |  |
| Web of Science | Science Citation Index Expanded (1900 to present) | No | Title abs author keywords | 3898 |
|  | Social Sciences Citation Index (1900 to present) |  |  |  |
| Web of Science | Conference Proceedings Citation Index – Science (1990 to present) | Yes | Title abs author keywords | 157 |
|  | Conference Proceedings Citation Index – Social Sciences and Humanities (1990 to present) |  |  |  |
| Proquest | Databases: health and medicine, science and tech, social sciences. Source types: reports, dissertations and theses, other sources, working papers, conference papers and proceedings | Yes | Title, abstracts and all subjects and indexing | 498 |
